# Editorial: Intelligence Support for Mentoring Processes in Higher Education (and Beyond)

**DOI:** 10.3389/frai.2022.935020

**Published:** 2022-06-13

**Authors:** Ralf Klamma, Miloš Kravčík, Viktoria Pammer-Schindler, Elvira Popescu

**Affiliations:** ^1^Informatik 5 (Database and Information Systems), RWTH Aachen University, Aachen, Germany; ^2^Educational Technology Lab, German Research Center for Artificial Intelligence, Berlin, Germany; ^3^Department of Computer Science and Biomedical Engineering, Graz University of Technology, Graz, Austria; ^4^Computers and Information Technology Department, University of Craiova, Craiova, Romania

**Keywords:** mentoring process, mentoring, chatbots, deep learning, mixed reality, mentoring support, mentoring analytics, intelligent systems

Mentoring is the activity of a senior person (the mentor) supporting a less experienced person (the mentee) in learning. It is based on a trustful, protected, and private atmosphere between the mentor and the mentee. The goal is to develop a professional identity and to reflect on the current situation. At universities, mentors are senior academics or skilled employees while mentees are mostly students with various competencies. Outside universities, mentors and mentees are professionals. In technology-enhanced learning, there is a longstanding tradition of supporting relationships between learners and teachers. Intelligent tutoring systems focus on the cognitive aspects of learning in a selected domain. They have been successfully applied, especially in such areas where the domain of knowledge can be well-formalized with the help of experts. Nevertheless, the learning process is also affected by motivations, emotions, and meta-cognitive competencies, which play a crucial role. In recent studies, these have been recognized and monitored through big educational data and a wide spectrum of available sensors. This enables support for the mentoring process, which is usually spontaneous, holistic, and depends on the needs and interests of the mentee. Psychological and emotional support are at the heart of the mentoring relationship, underpinned by empathy and trust.

This Research Topic aimed to investigate relevant aspects of mentoring processes and how they can be technologically supported. The four accepted papers offer various perspectives, ranging from a conceptual analysis of ethical questions to research contributions dealing with concrete scalability tools, novel teaching algorithms as well as policy recommendations in pandemic situations.

Köbis and Mehner discuss the relevant ethical questions involved with mentoring and in this way raise awareness of the ethical development and use of future data-driven AI-supported mentoring environments in higher education. They have juxtaposed principles of mentoring ethics and AI ethics with the objective of raising awareness in this interdisciplinary field.

Neumann et al. investigate how personal mentoring can be made scalable. They describe the development and implementation of two chatbots that aim to support students of the educational sciences in the self-study of seminar topics and literature. Their results from real-world experiences have the potential to improve the availability of digital mentoring support for all students.

Melo and Lopes propose the first machine teaching algorithm for multiple inverse reinforcement learners. Their theoretical analysis shows that teaching a sequential task to a heterogeneous class of learners with a single demonstration may not be possible, as the differences between individual agents increase. They contribute two algorithms that address the main difficulties identified.

Batucan et al. explain the factors affecting online learning amidst the COVID-19 pandemic. They empirically test the proposed extended unified theory of acceptance and use of technology (e-UTAUT) model in the students' intention and use behavior toward the online learning system. Insights for higher education institutions and policy directions are recommended.

## Author Contributions

All authors listed have made a substantial, direct, and intellectual contribution to the work and approved it for publication.

## Conflict of Interest

The authors declare that the research was conducted in the absence of any commercial or financial relationships that could be construed as a potential conflict of interest.

## Publisher's Note

All claims expressed in this article are solely those of the authors and do not necessarily represent those of their affiliated organizations, or those of the publisher, the editors and the reviewers. Any product that may be evaluated in this article, or claim that may be made by its manufacturer, is not guaranteed or endorsed by the publisher.

